# Reproducibility and validity of portable haemoglobinometer for the diagnosis of anaemia in children under the age of 5 years

**DOI:** 10.1017/jns.2019.43

**Published:** 2020-01-20

**Authors:** Alessandra da Silva Pereira, Inês Rugani Ribeiro de Castro, Flávia Fioruci Bezerra, José Firmino Nogueira Neto, Ana Carolina Feldenheimer da Silva

**Affiliations:** 1 Departamento de Nutrição Fundamental, Universidade Federal do Estado do Rio de Janeiro, Rio de Janeiro, Brazil; 2 Departamento de Nutrição Social, Universidade do Estado do Rio de Janeiro, Rio de Janeiro, Brazil; 3 Departamento de Nutrição Básica e Experimental, Universidade do Estado do Rio de Janeiro, Rio de Janeiro, Brazil; 4 Laboratório de Lipídeos/Policlínica Piquet Carneiro/UERJ, Rio de Janeiro, Brazil

**Keywords:** Children, Anaemia, Reproducibility, Accuracy, ICC, intraclass correlation coefficient, NPV, negative predictive value, PABAK, prevalence-adjusted bias-adjusted κ, PPV, positive predictive value

## Abstract

Portable haemoglobinometers have been used in order to estimate the prevalence of anaemia in diverse settings. However, few studies have been conducted to evaluate their performance in children of different age groups in distinct epidemiological contexts. To evaluate the reproducibility and reliability of a portable haemoglobinometer for the diagnosis of anaemia in children <5 years Hb was measured in the venous blood of 351 children <5 years by an automated system (standard method) and in three capillary blood samples, using a portable haemoglobinometer (HemoCue^®^; test method). The reproducibility of the device and of the test method was evaluated using the intraclass correlation coefficient (ICC) (Hb in its continuous form), κ and prevalence-adjusted bias-adjusted κ (PABAK) (categorised variable: anaemia: yes/no). For test method validation, Bland–Altman analyses were performed and sensitivity, specificity, accuracy rate, positive predictive value (PPV) and negative predictive values (NPV) were calculated. The haemoglobinometer presented good device reproducibility (ICC = 0·79) and reasonable method reproducibility (puncture, collection and reading) (ICC = 0·71). Superficial and fair agreement (κ) and good agreement (PABAK) were observed among the diagnoses obtained through the test method. The prevalence of anaemia was 19·1 and 19·7 % using the standard and the test method, respectively, with no statistically significant differences. The test method presented higher specificity (87·7 %) and NPV (88·3 %) than sensitivity (50·7 %) and PPV (49·3 %), and intermediary accuracy rate (57·8 %). HemoCue^®^ showed good device reproducibility and reasonable method reproducibility, as well as good performance in estimating the prevalence of anaemia. Nevertheless, it showed a fair reliability and low individual diagnostic accuracy.

Fe-deficiency anaemia, the final stage of Fe deficiency, is a condition which can make an impact on children's health and development^(1–3)^. High Fe-deficiency anaemia prevalence has had an impact on both health and the economy in countries around the world; therefore much effort has been put into the prevention and treatment of this blood disorder in the last few decades^(3)^.

Anaemia can result from many conditions, including acute/chronic inflammation, infectious diseases and haematological disorders, as well as insufficient intake of folate, vitamin B_12_ or, most commonly, Fe^(2,3)^. In this way, it is vital to appropriately estimate the prevalence of Fe-deficiency anaemia to inform the planning and evaluation of public policies. Thus, it is essential that an appropriate diagnostic method be adopted, since its performance can influence the ability to estimate the prevalence of this condition.

Usually, anaemia diagnosis is based on the blood concentration of Hb (referred to as ‘Hb’), which can be analysed by different methods^(4–6)^. The standard method is the analysis of venous blood Hb according to its conversion into cyanmethaemoglobin^(7)^. Another method employed is the analysis of Hb by automated cell counters, which can use different reagents^(8–10)^. Both methods depend on venous blood collection, which is invasive, and are both costly and complex to operationalise. Epidemiological studies have used cheaper, less invasive and more practical methods for anaemia diagnosis, such as portable haemoglobinometers. This equipment analyses Hb based on azide methaemoglobin concentration and its immediate measurement can be performed with capillary blood samples^(5,11,12)^.

Studies assessing the reproducibility and reliability of portable haemoglobinometers in determining Hb concentrations and estimating anaemia prevalence have been carried out since the 1980s^(4,9,13–25)^. However, considerable heterogeneity has been identified with regard to the age range of the group studied (i.e. children, adults), the type of blood sample analysed (venous or capillary blood), the standard method adopted (automated cell counters, cyanmethaemoglobin) and the performance assessment used (reliability). Eight studies included children^(9,15,17,18,22–25)^; among them, five assessed the performance of HemoCue^®^ in determining Hb based on capillary blood samples compared with a standard method with venous blood samples^(17,22–25)^. None of them compared children below 2 years of age with the older ones.

Despite some studies suggesting good performance by the haemoglobinometers, it is important to consider that reliability and reproducibility are not inherent to a certain method: its performance is dependent on the study's context^(26)^. Two aspects that could differ among the settings and influence the method's performance are: the age range of the group studied (assuming that the method's performance depends on the calibre of the vessels from where the capillary blood samples are collected) and the prevalence/level of severity of the anaemia (assuming that the method's performance can be different depending on Hb level/distribution).

In settings where the prevalence of anaemia varied between 20 and 50 % studies found good correlation between HemoCue^®^ and the standard method^(17,22)^. On the other hand, in those with higher (above 65 %)^(25)^ or lower (below 15 %)^(24)^ prevalence rates, the method's performance was worse.

The objective of the present study was to assess the reproducibility and reliability of a portable haemoglobinometer in diagnosing anaemia in children <2 and ≥2 years, users of the public health system in Brazil's second largest city in a context of mild prevalence of anaemia (13·7 %), according to the WHO^(27)^. More information on reproducibility and reliability of portable haemoglobinometers in young children will contribute to the assessment of their performance in settings potentially different from those where previous studies with young children were carried out.

## Materials and methods

### Public health problem definition

Anaemia is defined as the condition in which the Hb content in the blood is below normal as a result of the lack of one or more essential nutrients such as Fe, Zn, vitamin B_12_ and protein. However, Fe-deficiency anaemia is much more common than the others. The prevalence of anaemia as a public health problem can be categorised: (a) no public health problem when prevalence of anaemia is <5 %; (b) mild public health problem when prevalence of anaemia is 5–19·9 %; (c) moderate public health problem when prevalence of anaemia is 20–39·9 %; and (d) severe public health problem when prevalence of anaemia is ≥40 %^(3)^.

### Study design, population and sample

A cross-sectional study was carried out on a probabilistic sample of children aged 6–59 months, users of the Brazilian public health system (Sistema Único de Saúde; SUS) in the municipality of Rio de Janeiro who were enrolled in the ‘Anemia and Vitamin A deficiency in preschool children: prevalence in a major urban centre and validation of diagnostic methods’ survey.

Children were excluded from the study if they presented infectious diseases, such as pneumonia and otitis, among others, as well as sickle cell anaemia and hepatopathies. To calculate the sample size, the following parameters were considered: anaemia prevalence of 60·2 %, a value adopted based on a systematic review of studies carried out among health facilities^(5)^; correlation values resulting from previous studies that evaluated Hb using portable haemoglobinometers^(9,13,16)^, with the most conservative being adopted; absolute study precision of 0·20 and 95 % reliability level. By applying these parameters, a total of 111 children was reached. The sample size was calculated using the Stata v.10 program.

Considering that: (a) part of the correlation values adopted as a parameter for calculating the sample size was not obtained from studies with children, and (b) these parameters were attained treating the variables of interest in their continuous form, while in the present study variables were also analysed categorically, the sample size was increased by 50 %, totalling 167 children. Assuming 30 % of refusals, at least 217 children should be invited to participate. Children were randomly selected maintaining the Sistema Único de Saúde (SUS) database 1·5 ratio between those over and under the age of 2 years.

The survey that originated this study included 536 children aged 6–59 months and revealed an anaemia prevalence of 13·7 %. The present study was carried out on a subsample of children who had Hb levels measured by both venous blood in an automated system and capillary blood in a portable haemoglobinometer (*n* 351; 158 <2 years and 193 ≥2 years).

This study was approved by the Rio de Janeiro Municipal Health Office Ethics Committee for Research with Humans (no. 203A/2013). Parents or legal guardians were duly informed about the risks and benefits of taking part in the study. The study was conducted only on children whose parents or guardians agreed to their participation and signed a free and informed consent form. Blood test results were returned by researchers directly to the guardians of the children studied on pre-scheduled dates. Children who presented any disorder were referred to the Health Unities where the study had been carried out. The survey was supported by the National Scientific and Technological Development Council (Conselho Nacional de Desenvolvimento Científico e Tecnológico (CNPq) 480804/2013-3) and by the municipality of Rio de Janeiro.

### Blood sample collection and Hb analysis

Data collection was carried out between July and December 2014. Venous and capillary blood samples were collected by trained technicians experienced in collecting children's blood samples.

#### Standard method

We collected 4 h fasting venous blood samples into 0·5 ml EDTA evacuated tubes. For children under 1 year old, the blood was collected with a syringe and immediately transferred into EDTA tubes. The tubes were transported to the clinical analysis laboratory in a cool box up to 4 h after collection for Hb analysis which was performed on the same day.

Hb concentration on venous blood samples was analysed by the automated XS1000i Sysmex^®^ counter through the standard sodium lauryl sulfate (SLS)-Hb) detection method.

#### Test method

After venous blood collection, a capillary puncture of the middle or ring finger (children ≥1 year) or heel (children <1 year) was performed using disposable lancets. Three samples of capillary blood were collected from each child. The first and second sample tests (sample test 1 and sample test 2) were collected from the same puncture for the analysis for HemoCue^®^ reproducibility. The third sample test (sample test 3) was obtained from a second puncture performed on another finger or on another region of the heel in order to evaluate method reproducibility. According to the portable haemoglobinometer manufacturer recommendations, the first drop was discarded for each puncture.

Hb concentration in capillary blood samples was carried out by a portable HemoCue^®^ Hb 301 model haemoglobinometer by the azide methaemoglobin detection method (test method)^(13)^.

### Data analysis

To describe the group studied, the following variables were considered: age, sex, nutritional status (according to height-for-age and BMI-for-age), according to WHO classification criteria^(28)^ and maternal level of education. Nutritional status assessment was carried out using WHO Anthro Software^(29)^.

Hb concentration was considered the outcome variable, which was analysed in its continuous as well as categorical form (anaemic *v.* non anaemic). Children were considered anaemic if their Hb levels were below 11·0 g/dl (6·8 mmol/l)^(3)^. The normality of the Hb distribution was confirmed through Kolgomorov–Smirnov.

To describe the outcome in the group studied, Hb distributions obtained by both methods were presented in graphs and central tendency (means and medians) and dispersion (standard deviations, variances and interquartile ranges) measures were estimated. The statistical program SPSS version 17.1 was used for data analysis.

### Reproducibility assessment of the measures

The reproducibility of the HemoCue^®^ device was assessed by checking the consistency of the measures between sample test 1 and sample test 2, obtained from the same puncture of the children's finger or heel. The reproducibility of the method was assessed by checking the consistency of the measures between sample test 1 and sample test 3.

Assessment of reproducibility of both the device and method was performed using the intraclass correlation coefficient (ICC) to analyse the correlation between the results of the continuous variable (Hb concentration), assuming that values above 0·70 indicate good correlation^(30)^. For the variable in its categorical form (anaemia yes/no), κ coefficient and prevalence-adjusted bias-adjusted κ (PABAK)^(31)^ were used, since the κ coefficient is influenced both by the event prevalence and the measurement bias. The agreement values for κ and PABAK were interpreted by the classification proposed by Byrt^(32)^: −1·00 to 0·00: no agreement; 0·01 to 0·20: poor; 0·21 to 0·40: superficial; 0·41 to 0·60: fair; 0·61 to 0·80: good; 0·81 to 0·92: very good; and 0·93 to 1·00: excellent.

### Portable haemoglobinometer reliability assessment

Assessment of the test method accuracy was carried out by comparing the mean Hb values and anaemia prevalence obtained from HemoCue^®^ with those results obtained from the standard method. Comparisons between mean Hb values were conducted by paired Student's *t* test and comparisons between prevalence ratios were conducted using the McNemar χ^2^ test, adopting a 0·05 significance level. The agreement between Hb values obtained by the standard and test methods was examined using Bland–Altman analyses.

Sensitivity, specificity, accuracy index, positive predictive value (PPV) and negative predictive value (NPV) of the test method were also calculated in order to evaluate the test method accuracy in the individual anaemia diagnosis^(30,33)^.

## Results

### Population characteristics

Among the 351 children who took part in the study, 51·0 % were females and 45 % were aged between 6 and 23 months. The mean age was 16·0  (sd) 5·0 months for children <2 years and 41·6 (sd 10·5) for those ≥2 years. Concerning the maternal level of education, 52·1 % had completed elementary school, 44·0 % had concluded high school education and 3·9 %, higher education. These results were similar for both age groups studied. With regard to the nutritional status, 4·9 % had low height for age, 23 % were at risk of overweight, 5·6 % were overweight and 2·5 % were obese (BMI/age). The prevalence of anaemia was 19·1 % for the total group, being 32·9 and 7·8 % for children <2 and ≥2 years, respectively.

### Device and method reproducibility

**Fig. 1.**
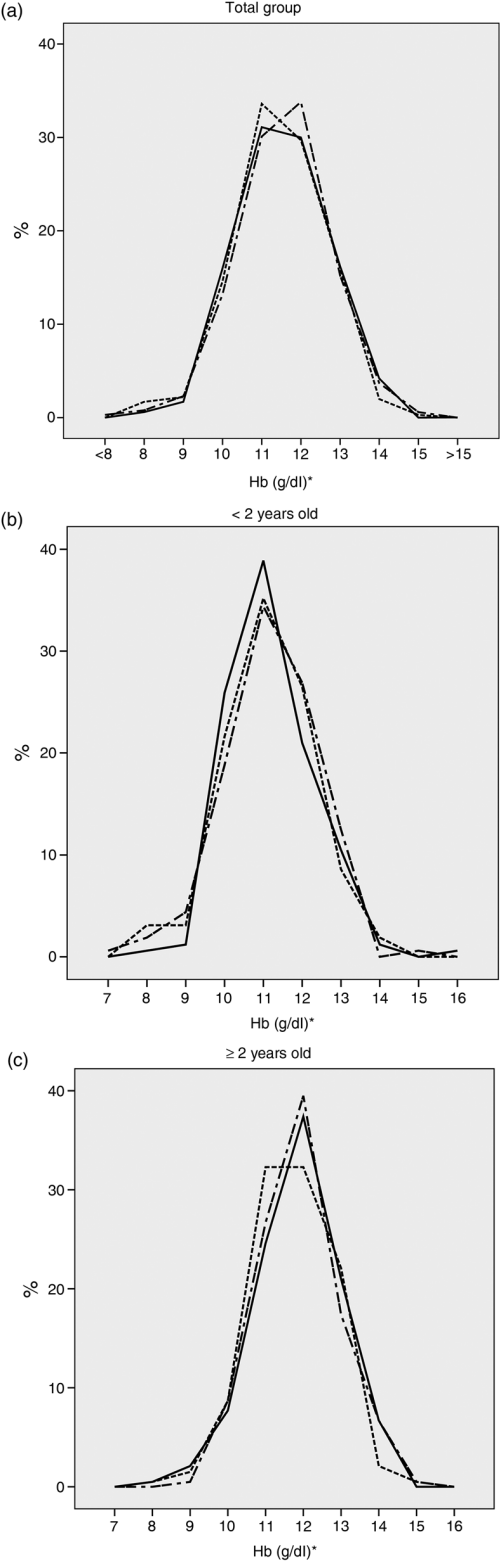
Distribution of Hb concentration of three capillary blood samples obtained through a portable haemoglobinometer in children aged 6 to 59 months (Rio de Janeiro, 2014). *n* 357 (sample test 1 (––) *v.* sample test 2 (---)) and *n* 355 (sample test 1 *v.* sample test 3 (– –)). Sample test 1: first drop of capillary blood, extracted from the first puncture, read on a portable haemoglobinometer. Sample test 2: second drop of capillary blood, extracted from the first puncture, read on a portable haemoglobinometer. Sample test 3: third drop of capillary blood, extracted from the second puncture, read on a portable haemoglobinometer. (a) Total group; (b) < 2 years old; (c) ≥ 2 years old. * To convert Hb in g/dl to mmol/l, multiply by 0·6206.

The distributions of Hb values were similar between the three test method samples for both the total group studied and by age range (Fig. 1).

According to the ICC, the agreement observed between sample tests 1 and 2 was good (varying between 0·75 and 0·81), and a little higher than that observed between sample tests 1 and 3 (which varied between 0·68 and 0·71) (Table 1). When analysing Hb as a categorical variable, agreement was fair between sample test 1 and sample test 2 (κ = 0·43) and superficial between test 1 and test 3 (κ = 0·36) for the total group, with better results observed for children <2 years (κ = 0·46 and 0·42, respectively) compared with those observed for older children (κ = 0·28 and 0·18, respectively). When using PABAK, agreement was good (varying between 0·61 and 0·69), except for sample tests 1 and 2 among children <2 years, which remained fair (0·54) (Table 1).

**Table 1.** Hb concentration, intraclass correlation coefficient, κ and adjusted κ for Hb determination and diagnosis of anaemia by portable haemoglobinometer in children aged 6 to 59 months by age group (Rio de Janeiro, 2014)*

	Hb (g/dl)†		Intraclass correlation coefficient		
Test method	Mean	sd		κ	Adjusted κ
Children <2 years				
Sample test 1‡	11·70	1·06	–	–	–
Sample test 2§	11·57	1·16	0·81	0·46	0·54
Sample test 3ǁ	11·67	1·15	0·68	0·42	0·61
Children ≥2 years					
Sample test 1	12·31	1·08			
Sample test 2	12·18	1·06	0·75	0·28	0·69
Sample test 3	12·28	0·98	0·68	0·18	0·67
Total					
Sample test 1	12·04	1·11	–	–	–
Sample test 2	11·70	1·15	0·79	0·43	0·62
Sample test 3	12·00	1·10	0·71	0·36	0·63

* *n* 357 (test 1 *v.* test 2) and 355 (tests 1 *v.* 3). Test method: Hb concentration in capillary blood sample analysed by portable haemoglobinometer (HemoCue^®^ Hb 301).

† To convert Hb in g/dl to mmol/l, multiply by 0·6206.

‡ Sample test 1: 1st drop of capillary blood, extracted from the first puncture, read on a portable haemoglobinometer.

§ Sample test 2: 2nd drop of capillary blood, extracted from the first puncture, read on a portable haemoglobinometer.

ǁ Sample test 3: 3rd drop of capillary blood, extracted from the second puncture, read on a portable haemoglobinometer.

### Reliability of the portable haemoglobinometer

**Fig. 2.**
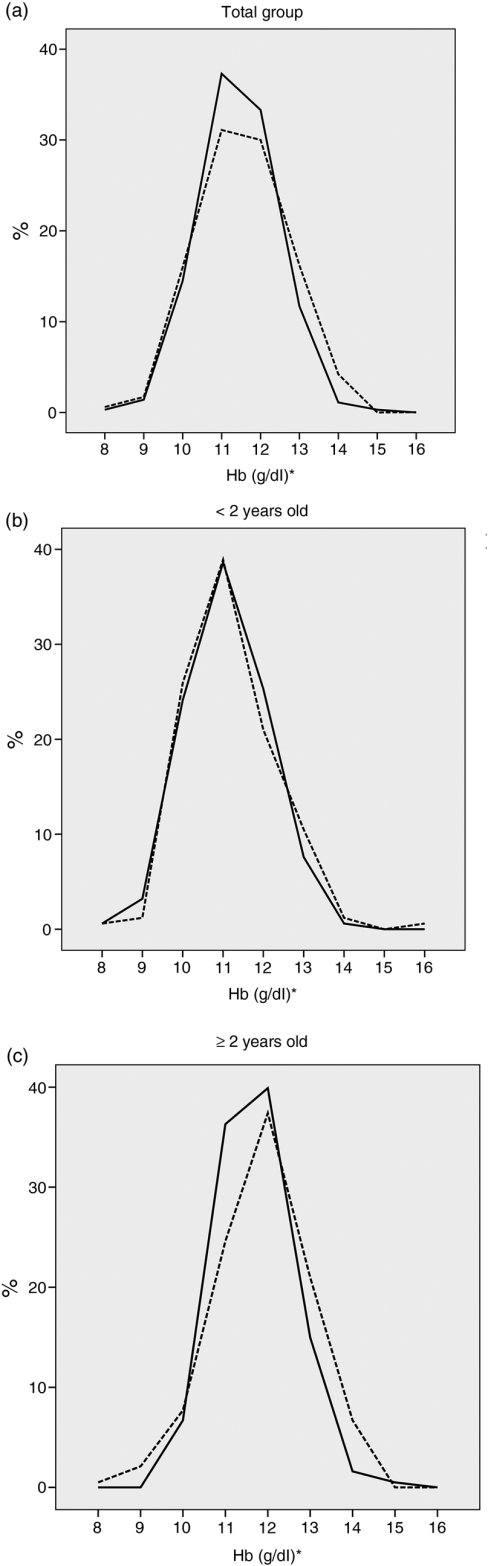
Distribution of Hb concentration according to the standard method (––) and the test method (---) carried out on children aged 6 to 59 months by age group (Rio de Janeiro, 2014). *n* 351. Standard method: Hb analysis in venous blood samples by automated system (XS1000i Sysmex^®^). Test method: Hb concentration in capillary blood sample analysed by portable haemoglobinometer (HemoCue^®^ Hb 301). (a) Total group; (b) < 2 years old; (c) ≥ 2 years old. * To convert Hb in g/dl to mmol/l, multiply by 0·6206.

The distribution of Hb values obtained by standard and test methods resulted in considerably overlapped curves, with values slightly higher for the test method for both the total group (Fig. 2(a)) and by age range (Fig. 2(b) and 2(c)).

**Fig. 3.**
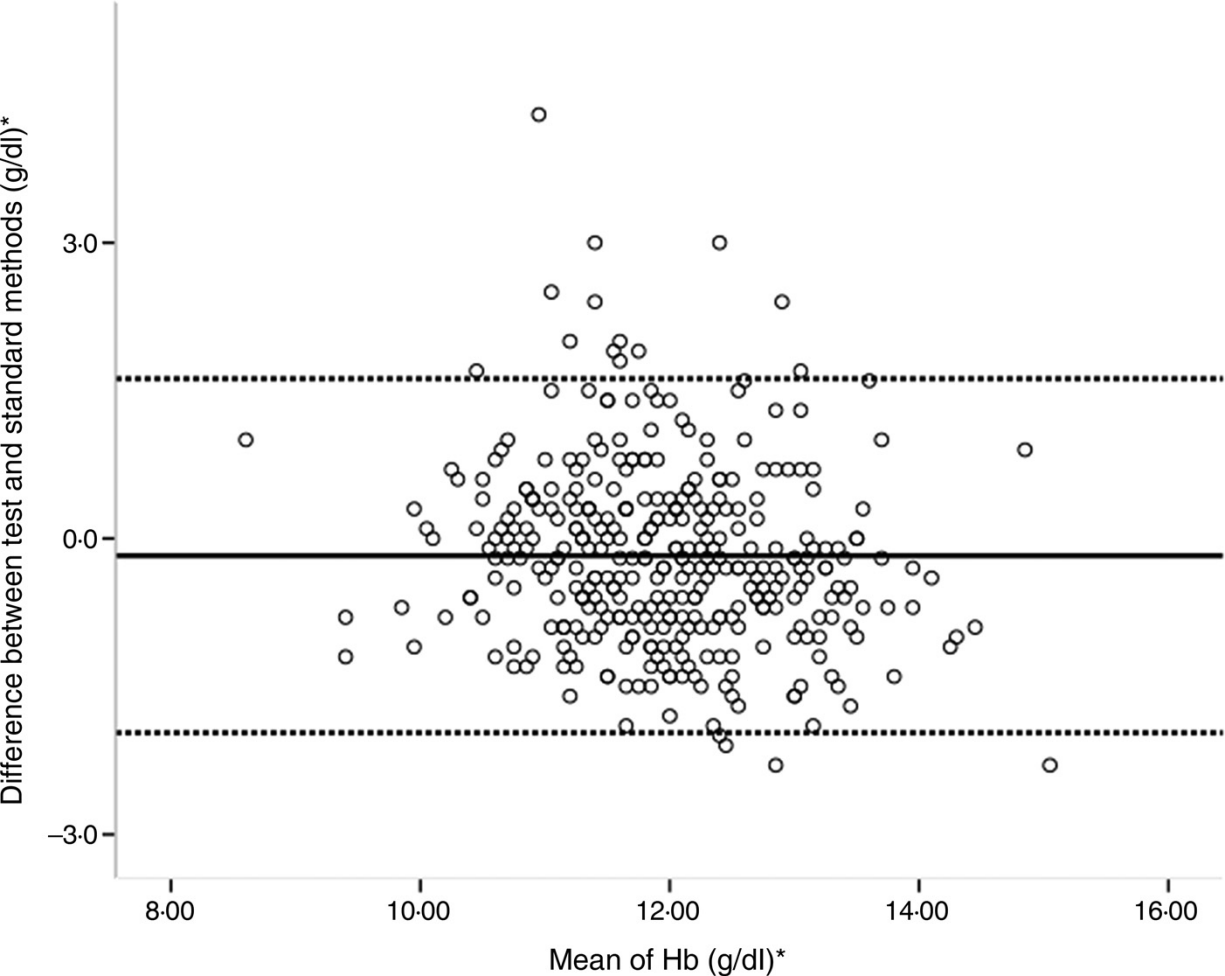
Bland−Altman plot showing agreement in Hb concentration of children aged 6 to 59 months of Rio de Janeiro assessed by test method (Hb concentration in capillary blood sample analysed by portable haemoglobinometer: HemoCue^®^ Hb 301) and standard method (Hb analysis in venous blood samples by automated system: XS1000i Sysmex^®^). * To convert Hb in g/dl to mmol/l, multiply by 0·6206.

For the total group, Hb mean values obtained through the standard method and the test method (respectively, 11·86 and 12·04 g/dl (7·36 and 7·47 mmol/l)) showed a statistically significant difference (*P* < 0·001), although the magnitude of that difference was not very large (0·18 g/dl (0·11 mmol/l)) (Table 2). A similar result was observed for both age groups. A Bland−Altman plot shows that the mean difference between Hb values estimated by the test and standard methods was –0·17 g/dl (–0·11 mmol/l), with 95 % limits of agreement from −1·98 to 1·62 g/dl (–1·23 to 1·01 mmol/l) (Fig. 3).

**Table 2.** Hb concentration and prevalence of anaemia according to the standard method and the test method in children aged 6 to 59 months (*n* 351) by age group (Rio de Janeiro, 2014)

Method	Hb (g/dl)*					Prevalence of anaemia (%)
	Mean	sd	Median	Variance	Interquartile range	
Children <2 years						
Standard†	11·52	0·99	11·50	1·00	1·40	32·9‡
Test§	11·70	1·06ǁ	11·70	1·13	1·40	29·1‡
Children ≥2 years						
Standard†	12·14	0·87	12·10	0·75	1·20	7·8‡
Test§	12·31	1·08ǁ	12·40	1·18	1·60	11·9‡
Total						
Standard†	11·86	0·97	11·80	0·95	1·30	19·1‡
Test§	12·04	1·11ǁ	12·00	1·25	1·50	19·7‡

* To convert Hb in g/dl to mmol/l, multiply by 0·6206.

† Standard method: Hb concentration in venous blood sample analysed by automated system (XS1000i Sysmex^®^).

‡ No statistically signficant difference (*P* > 0·05) between prevalences of anaemia obtained by the standard method and the test method (McNemar's χ^2^ test).

§ Test method: Hb concentration in capillary blood sample analysed by portable haemoglobinometer (HemoCue^®^ Hb 301).

ǁ Statistically significant difference (*P* < 0·05) between the mean Hb concentration obtained by the standard method and the test method (Student's *t* test for paired samples).

No statistically significant differences were observed for the anaemia prevalence obtained by both the standard and test methods for the total group (19·1 and 19·7 %, respectively), children <2 years (32·9 and 29·1 %, respectively) and older children (7·8 and 11·9 %, respectively) (Table 2).

Regarding the performance of the test method in the individual diagnosis of anaemia, it was observed that for the total group studied as well as for children in each age group (<2 and ≥2 years), it showed higher specificity (87·7, 76·7 and 95·9 %, respectively) than sensitivity (50·7, 56·5 and 34·8 %, respectively), suggesting more accuracy in false positive exclusion. The test method also showed higher NPV (88·3, 81·1 and 91·6 %, respectively) than PPV (49·3, 50·0 and 53·3 %, respectively). The accuracy index was 57·8 % for the total group studied, with quite distinct results within each age range (73·6 and 36 % for <2 and ≥2 years, respectively) (Table 3).

**Table 3.** Sensitivity, specificity, accuracy index, positive predictive value and negative predictive value of the portable haemoglobinometer compared with the automated system for estimating anaemia in children aged 6 to 59 months according to age group (Rio de Janeiro, 2014)

Age group	Sensitivity (%)	Specificity (%)	Accuracy index (%)	Positive predictive value (%)	Negative predictive value (%)
Children <2 years (*n* 158)	56·5	76·7	73·6	50·0	81·1
Children ≥2 years (*n* 193)	34·8	95·9	36·3	53·3	91·6
Total (*n* 351)	50·7	87·7	57·8	49·3	88·3

## Discussion

The test method showed good reproducibility when considering Hb as a continuous variable and fair reproducibility for Hb in its categorical form. When compared with the standard method, it showed higher Hb averages, similar anaemia prevalence estimates, low PPV and sensitivity, high NPV and specificity and intermediate accuracy index.

The comparison of these results with those from other publications is limited given the difference, among the studies, in the age range of the group analysed, the type of blood samples used (resulting from capillary and/or venous blood), the standard method used (cyanmethaemoglobin or azide methaemoglobin) and the statistical tests used. Nevertheless, it can be observed that other studies also verified higher Hb concentration in capillary blood analysed by portable haemoglobinometer when compared with that measured in a venous blood sample. In a study carried out from 2000 to 2002 with 413 children from Kansas and 213 from St Louis, USA, with ages varying from 12 to 35 months, Boghani *et al.*^(24)^ observed higher Hb concentrations in capillary blood analysed using HemoCue^®^ (12·5 and 11·6 g/dl (7·8 and 7·2 mmol/l), respectively) when compared with those obtained from venous blood analysed by the automated system (11·8 and 11·5 g/dl (7·3 and 7·1 mmol/l), respectively), with no statistically significant difference.

A converging result was obtained in a study conducted in India with 100 children aged 1–6 years which compared capillary blood Hb concentrations analysed by HemoCue^®^ with venous blood analysed by the cyanmethhaemoglobin method. In that study, a larger (1·19 g/dl (0·7 mmol/l)) and statistically significant difference in between methods was observed^(18)^.

On the other hand, Levy *et al*.^(23)^ performed a study with 137 children aged 1–5 years in San Luis Potosí, Mexico, in 2015, and found a lower mean capillary blood Hb level (12·7 g/dl (7·9 mmol/l), analysed using HemoCue^®^) when compared with that obtained from venous blood (14·2 g/dl (8·8 mmol/l)) analysed by the automated system, with a statistically significant difference.

Studies using HemoCue^®^ to assess Hb concentration from a venous blood sample showed higher concentrations when compared with the blood samples analysed by an automated system or by cyanmethaemoglobin^(15,22)^. On the other hand, in a study carried out in Morelos, Mexico, with seventy-two children aged 6 months–15 years a higher Hb concentration was observed by the automated system both with capillary and venous blood samples, when compared with HemoCue^®(17)^.

The difference between Hb concentration in venous and capillary blood is shown in other studies and is biologically plausible^(21,34–36)^. Patel *et al*.^(21)^ suggest that the contents of a drop of capillary finger blood reflect the blood contents of different finger capillaries, which can alter the determination of Hb concentration. Furthermore, the authors suggest that the capillary blood sample from the finger depends on skin thickness and temperature, the depth of the lancet penetration and the finger blood extraction technique performed by the professional. Furthermore, Hagan *et al*.^(37)^ emphasise the importance of body positioning at the time of collection, since this may influence the Hb values in capillary blood; blood samples collected in the supine position produce higher results than those obtained in a vertical position.

In the present study, ICC values indicated good consistency for the portable haemoglobinometer (ICC above 0·70) in determining Hb concentrations with the same capillary puncture (comparison between sample tests 1 and 2) both for the total group and by age range. For capillary blood samples resulting from another puncture (comparison between sample tests 1 and 3), ICC values were very close to 0·70, suggesting good reproducibility for HemoCue^®^. In a study involving eighty-seven females, Morris *et al*.^(15)^ observed reasonable haemoglobinometer reproducibility for determining Hb in capillary blood samples at different sites (left and right hand fingers; ICC = 0·69), and fair reproducibility in capillary blood samples from the same person on 4 consecutive days (ICC = 0·50). Paiva *et al*.^(38)^, on the other hand, observed fair reproducibility (ICC = 0·60) of HemoCue^®^ in determining Hb in the capillary and venous blood samples of twenty-nine pregnant women.

On a complementary basis, the CV of the results^(39)^ was also examined. CV values were relatively high, especially among children <2 years (10·2 % for test 2 and 9·8 % for test 3), when compared with those from other studies with children. In the study conducted by Morris *et al*.^(15)^ with eighty-seven woman from Honduras and seventy-three from Central Bangladesh, the CV in determining Hb from two different sites (one in each hand, study from Honduras) in the same day was 6·3 % and in 4 consecutive days (study from Bangladesh) was 7·0 %. In a study conducted with twenty-nine children with ages ranging from 15 to 32 months, in Khammouane province, Laos, in 2016, Hinnouho *et al*.^(25)^ compared the venous blood Hb values obtained by three different portable haemoglobinometer devices and observed CV values ranging from 6·1 to 8·0 among them. Paiva *et al*.^(38)^, in a study with twenty-nine pregnant women, observed the CV for the determination of Hb values analysed by the haemoglobinometer with capillary blood (CV = 13·6) and venous blood samples (CV = 17). Von Schenck *et al*.^(13)^ observed good precision from HemoCue^®^ (CV = 1·3 %) in determining Hb when they analysed fifty-two pairs of capillary blood samples from young adults of both sexes.

In the haemoglobinometer reproducibility analysis for Hb in its categorical form, κ values showed that the haemoglobinometer showed better performance in capillary blood samples from the same puncture (sample tests 1 and 2) when compared with samples from the second puncture (sample tests 1 and 3), in both age groups, showing better results among the younger children. PABAK values indicated the influence of prevalence in the present findings. Calculating the contribution of the bias component within this dataset, it was observed that it did not influence the PABAK result. By comparing both age groups, it was possible to observe that among the children under 2 years of age, who presented higher anaemia prevalence, the difference observed between the κ and PABAK values was smaller. These results reiterate the assertion that reproducibility is not a characteristic inherent to the method, and reinforce the importance of evaluating the performance of the haemoglobinometer in different scenarios. We did not find in studies that κ and/or PABAK was used to analyse the reproducibility of the portable haemoglobinometer, which limited the comparison of our results with the literature.

Regarding the reliability, in this study the haemoglobinometer presented superficial reliability and showed better performance in detecting the individual situation of absence of anaemia. Converging results to this were registered by Boghani *et al*.^(24)^, who observed sensitivity of 32·8 and 60·4 % and specificity of 97·7 and 85·6 % between the children from Kansas and those from St Louis, respectively. Hinnouho *et al*.^(25)^ evaluated the reliability of the portable haemoglobinometer for the determination of anaemia comparing capillary and venous blood samples, from 1487 Laotian children, analysed by an automated system and observed a sensitivity of 68·7 % and specificity of 85·8 %.

It is worth mentioning that both studies mentioned showed a similar design to that in the present study; in other words, comparison of the portable haemoglobinometer in determining anaemia based on Hb concentration in capillary blood samples with that obtained from a venous blood sample and analysed by an automated system as standard method.

Other studies, however, have shown a better ability of the haemoglobinometer to detect true anaemics. It is worth highlighting, however, that in these cases the participants' age group and the blood sample analysed by the test method (venous blood) were different from those adopted here. In a study carried out with 184 children aged from 72 to 102 months using venous blood samples both in the test and standard methods, Gwetu & Chhagan^(22)^ assessed the ability of the HemoCue^®^ device to detect anaemia and observed sensitivity of 93 % and specificity of 74·5 %. In the study conducted by Boghani *et al*.^(24)^, the authors also evaluated the haemoglobinometer performance with venous blood samples in a group of children from Kansas and observed sensitivity of 51·7 %, higher, therefore, than that obtained from capillary blood samples (32·8 %). In a study with 320 individuals aged 18–63 years attending a blood donation centre, Shahshahani *et al*.^(40)^ evaluated the method's sensitivity and specificity on individuals with a high mean Hb (≥18 g/dl (≥11·2 mmol/l)) and low mean Hb (<12·5 g/dl (<7·8 mmol/l)) and observed, in the first group, sensitivity and specificity of 96·3 and 85·7 % for venous blood samples and 100 and 79·8 % for capillary blood samples, both analysed by portable haemoglobinometer. The authors observed that in the group with lower Hb levels, sensitivity dropped and specificity rose for both types of blood samples analysed.

In the present study, among the children <2 years, the group in which a higher prevalence of anaemia was observed (32·9 %), we found low sensitivity, low PPV and high NPV values. The opposite was found in children ≥2 years, who showed low anaemia prevalence (7·8 %). In the study conducted by Neufeld *et al*.^(17)^, in which a prevalence of 41·9 % of anaemia was found among infants and adolescents aged 6 months to 15 years, and in which HemoCue^®^ performance was assessed through capillary blood samples, the following values were found: sensitivity 84 %, specificity 93 %, PPV 90 % and NPV 89 %. As previously reported, Shahshahani *et al*.^(40)^ found that among adult blood donors, in the group showing lower capillary blood Hb concentration, HemoCue^®^ was less sensitive than specific, when compared with the one with higher Hb concentrations.

In a study carried out with 500 platelet donors in which a prevalence of 5·8 % of anaemia was found, Malhi *et al*.^(41)^ found a sensitivity of 50 % using HemoCue^®^ in the detection of anaemia using capillary blood samples when compared with venous blood samples analysed by an automated system, while specificity was 98·5 %. PPV and NPV were 70·8 and 96·4 %, respectively.

A strength of our study was the number of children studied, which allowed investigating the test method performance in children under 24 months and between 24 and 59 months of age. HemoCue^®^ showed greater stability in children <2 years. Lower Hb concentration, expected in younger children, could be one of the reasons for this finding. Knowing the performance of the portable haemoglobinometer on children under the age of 5 years in a context where anaemia prevalence was lower than those generally found in similar studies also contributed towards the innovative character of the present study.

Another strength of the study was the use of the κ and PABAK measurements, as well as ICC, to evaluate the reproducibility of HemoCue^®^, suggesting that it is important to assess HemoCue's^®^ reproducibility not only regarding the determination of Hb concentration, but also in relation to the detection (diagnosis) of the problem to be studied. The use of PABAK enabled knowing the influence of the prevalence of the event of interest in the κ index. A third positive aspect of the study was the analysis of the portable haemoglobinometer's reliability for diagnosing anaemia as well as for estimating the prevalence of this disease. In other words, we provided data on its performance for both individual and collective diagnosis, differing from many of the studies cited, which frequently did not present any prevalence data.

A weak point of the study to be mentioned is the fact that no analyses of the portable haemoglobinometer were carried out with venous blood samples, which would allow for the ascertainment of the haemoglobinometer's differences in determining Hb in both blood sample types. The decision to analyse only capillary blood samples was due to the fact that, in practice, HemoCue^®^ is used in epidemiological studies with such type of samples.

### Conclusion

HemoCue^®^ showed good device reproducibility and reasonable method reproducibility, as well as good performance in estimating the prevalence of anaemia. Nevertheless, it showed a fair reliability and low individual diagnostic accuracy, especially in the group of children ≥2 years, which presented low anaemia prevalence. Therefore, the utilisation of HemoCue^®^ appears to be adequate in population-based studies aimed at estimating the prevalence of anaemia among children, but not for the purpose of individual diagnosis, especially in contexts with low anaemia prevalence.
